# Parametric Cortical Representations of Complexity and Preference for Artistic and Computer-Generated Fractal Patterns Revealed by Single-Trial EEG Power Spectral Analysis

**DOI:** 10.1016/j.neuroimage.2021.118092

**Published:** 2021-04-23

**Authors:** Eric Rawls, Rebecca White, Stephanie Kane, Carl E. Stevens, Darya L. Zabelina

**Affiliations:** a Department of Psychiatry and Behavioral Sciences, University of Minnesota Health; b Department of Psychology, University of New Hampshire; c Department of Psychological Sciences, University of Arkansas

**Keywords:** Fractal, Power spectra, Perception, Preference, Aesthetic

## Abstract

Fractals are self-similar patterns that repeat at different scales, the complexity of which are expressed as a fractional Euclidean dimension D between 0 (a point) and 2 (a filled plane). The drip paintings of American painter Jackson Pollock (JP) are fractal in nature, and Pollock’s most illustrious works are of the high-D (~1.7) category. This would imply that people prefer more complex fractal patterns, but some research has instead suggested people prefer lower-D fractals. Furthermore, research has suggested that parietal and frontal brain activity tracks the complexity of fractal patterns, but previous research has artificially binned fractals depending on fractal dimension, rather than treating fractal dimension as a parametrically varying value. We used white layers extracted from JP artwork as stimuli, and constructed statistically matched 2-dimensional random Cantor sets as control stimuli. We recorded the electroencephalogram (EEG) while participants viewed the JP and matched random Cantor fractal patterns. Participants then rated their subjective preference for each pattern. We used a single-trial analysis to construct within-subject models relating subjective preference to fractal dimension D, as well as relating D and subjective preference to single-trial EEG power spectra. Results indicated that participants preferred higher-D images for both JP and Cantor stimuli. Power spectral analysis showed that, for artistic fractal images, parietal alpha and beta power parametrically tracked complexity of fractal patterns, while for matched mathematical fractals, parietal power tracked complexity of patterns over a range of frequencies, but most prominently in alpha band. Furthermore, parietal alpha power parametrically tracked aesthetic preference for both artistic and matched Cantor patterns. Overall, our results suggest that perception of complexity for artistic and computer-generated fractal images is reflected in parietal-occipital alpha and beta activity, and neural substrates of preference for complex stimuli are reflected in parietal alpha band activity.

## 1. Introduction

Fractals are self-similar patterns that repeat at different scales (Falconer, 2014; Mandelbrot, 1982). Mandelbrot, who was the first to characterize fractals, famously wrote: “Clouds are not spheres, mountains are not cones, coastlines are not circles, and bark is not smooth, nor does lightning travel in a straight line” (Mandelbrot, 1982). As alluded by this quote, fractals are prevalent in nature, ranging from sea anemones, to galaxies, to the structure of our DNA. Unlike conventional psychophysics and perception stimuli, fractals are non-Euclidian and structurally complex, with each of the parts being similar to the whole. This allows for insight into configural (as opposed to featural) processes such as natural scene perception (Zahedi & Zeil, 2018), spatial navigation (Juliani et al., 2016), Gestalt grouping principles (Farkas & Hajnal, 2013), and object recognition in computer vision and machine learning algorithms (Domenech et al., 2020; Tan & Yan, 1999, 2000; Wang et al., 2016) and in humans (Bies et al., 2016; Taylor et al., 2017). Fractals hold potential for such areas of research because their qualities (e.g. symmetry and proximity) yield the classic Gestalt principle of “figural goodness” (i.e. the degree to which a pattern can be organized into a coherent object). The geometric complexity of fractal patterns can be quantified by their dimension D, which falls between Euclidean dimensions. For images, the D parameter ranges between D = 1 (a line) and D = 2 (a filled plane). Dimensions closer to 2 indicate a higher degree of complexity, and generally cover a larger percentage of the plane (e.g., as pattern repetitions occur to a non-fractal line of D = 1, the D value moves closer to 2, and the line will occupy more space). However, it is possible for some 2D fractal images to have D < 1, when the patterns are sparse enough to constitute a point rather than a line (non-continuous or “dust” fractals) (Falconer, 2014). Given the controllability of complexity in fractal patterns and their relation to more general theories, fractals are particularly useful stimuli for research on human perception and aesthetic preference.

In the early 1940s, the abstract artist Jackson Pollock began creating his infamous splatter paintings by using a paintbrush to drip and fling paint onto long rectangles of yachting canvas. An analysis of Pollock’s splatter paintings confirmed that they have fractal characteristics (Taylor et al., 1999). Pollock’s technique evolved over time, consisting of a “preliminary phase” that yielded low-D fractal images (e.g. *Untitled*, 1945. D = 1.10), a “transitional phase” wherein resulting dimensions increased (e.g. *Number 14*, 1948, D = 1.45), and a “classic” period, where he mastered the technique and the D values of his works lingered around 1.7 (Taylor et al., 2002). During the classic period, he also painted *Untitled* (1950), where the complexity of his works peaked at D = 1.89. However, he quickly erased this design (which was painted on glass) and his following paintings scaled back again to D = 1.7. This suggests that he desired to generate fractal patterns with D ~ 1.7, spending nearly 10 years approaching, passing, then returning to this dimension (Taylor et al., 2007). There is some evidence that people aesthetically prefer fractals within the D range of 1.3 – 1.5 (as opposed to outside of this range) (Taylor et al., 2011; Viengkham & Spehar, 2018) [although see (Bies, Blanc-Goldhammer, et al., 2016)]. This would suggest that earlier works by Pollock, prior to his classic period, should have greater visual appeal. However, the paintings from his classic period remain as the most illustrious, suggesting that higher-D fractals might capture attention more efficiently than lower-D fractals. Taylor and colleagues (2011) speculate that Pollock found the “visually restful” mid-D range to be too simple and bland, and that he desired to keep his audience engaged with visually complex pieces.

Noninvasive neuroscience methods are a promising avenue to understand human perceptual and aesthetic responses to fractal patterns such as Pollock’s art. In particular, electroencephalography (EEG) is a powerful technique that yields precise timing of rhythms underlying cognitive processing. EEG is composed of activity that can be divided into delta (~1–3 Hz), theta (~4–8 Hz), alpha (~9–14 Hz), beta (~15–30 Hz) and gamma (~31 + Hz). Activity in different frequency bands supports stimulus coding, attention, and communication across neural ensembles (Lopes da Silva, 2013). Delta oscillations are related to motor demands in cognitive tasks (Harmony, 2013; Huster et al., 2013; Rawls et al., 2020; Stefanics et al., 2010) and to delivery of reinforcement-related outcomes (Bernat et al., 2015; Cavanagh, 2015; Rawls & Lamm, 2021), while theta oscillations are more generally related to the need for cognitive control (Cavanagh & Frank, 2014; Cohen & Cavanagh, 2011; Cooper et al., 2015; Nigbur et al., 2011). Alpha-frequency EEG broadly relates to internally-focused attention (Benedek et al., 2014; Knyazev et al., 2011; Ray & Cole, 1985), and has a role in creative experience (Stevens & Zabelina, 2019). Beta-frequency EEG is associated with sensorimotor brain activity including vision (Aissani et al., 2014; Battaglini et al., 2020; Gola et al., 2013; Piantoni et al., 2010) and motor output (Fischer et al., 2018; Schaller et al., 2017; Zaepffel et al., 2013). Finally, gamma oscillations are a marker of local neural ensemble synchronization, and might serve to bind together the features of a stimulus (Fries, 2009; Fries et al., 2007) – unfortunately, gamma and higher frequencies have low signal-to-noise ratio in noninvasive human EEG and are difficult to detect without optimized equipment (Cohen, 2014; Muthukumaraswamy & Singh, 2013).

Based on the aforementioned results, we might expect a role for beta frequencies in visual perception of fractal complexity and we might expect a role of alpha band activity in attention, evaluation, and aesthetic responses to fractal imagery. Only a few previous studies have used EEG to examine human brain activity during appraisal of fractal stimuli. Hagerhall and colleagues (Hagerhall et al., 2015) found that viewing mid-D statistical fractals (as opposed to exact fractals of the same dimension) produced the most alpha EEG activity. Hagerhall and colleagues (Hagerhall et al., 2008) found that fractal patterns of D = 1.3 produced the highest alpha and delta response in the frontal region, while con-currently producing the highest beta response in the parietal region. Several other studies have examined neural responses to fractal stimuli using fMRI (Isherwood et al., 2017; Rieger et al., 2013); however, fMRI techniques are unable to capture the fine temporal scale of perceptual brain processing due to the sluggish nature of the BOLD response.

In the present study, we recorded EEG while participants viewed and provided subjective ratings to statistically controlled artistic fractals (binarized Jackson Pollock images) and computer-generated fractals (random Cantor sets) with fractal dimension ranging parametrically from D = 1.10 to 1.89. We used a whole-scalp, single-trial modeling approach to examine brain activity that tracked the complexity of, and preference for, patterns within single subjects. Based on the illustriousness of Jackson Pollock’s high-D paintings, we hypothesized that participants would prefer artistic fractals with higher D to those with lower D. We predicted that alpha and beta activity over occipital and parietal cortex would track the complexity of fractal patterns, and that preference for fractal patterns would be reflected in alpha activity in frontal and parietal areas.

## 2. Methods

### 2.1. Participants

Fifty-one students at the University of Arkansas completed the pattern viewing study after providing informed consent (IRB #1902179495). Students were compensated for study completion with course credit in introductory psychology classes. Six participants were excluded due to data acquisition issues, and one additional participant was excluded for noncompliance (they gave the same subjective rating to every image), leaving forty-four participants for all analyses. See Table 1 for self-reported gender, age, and race demographics of the sample; included and excluded samples did not differ in gender, age, or race (all p >.2).

### 2.2. Stimuli and task

Participants completed a pattern viewing and subjective rating task while scalp EEG was recorded. During the viewing task, 90 binary JP images were presented, interspersed with 90 random Cantor fractals. All fractal stimuli subtended approximately 11° × 11° of visual angle. Subjects viewed each of 180 patterns for 4 seconds each, then rated each image based on personal preference for the pattern using keyboard keys 1 (*dislike*) to 9 (*like*). A fixation cross lasting between 2 and 3 seconds (jittered) was presented between each stimulus (patterns and rating screens). The fractal viewing and rating task required approximately 45 minutes to complete.

**Fig. 1.**
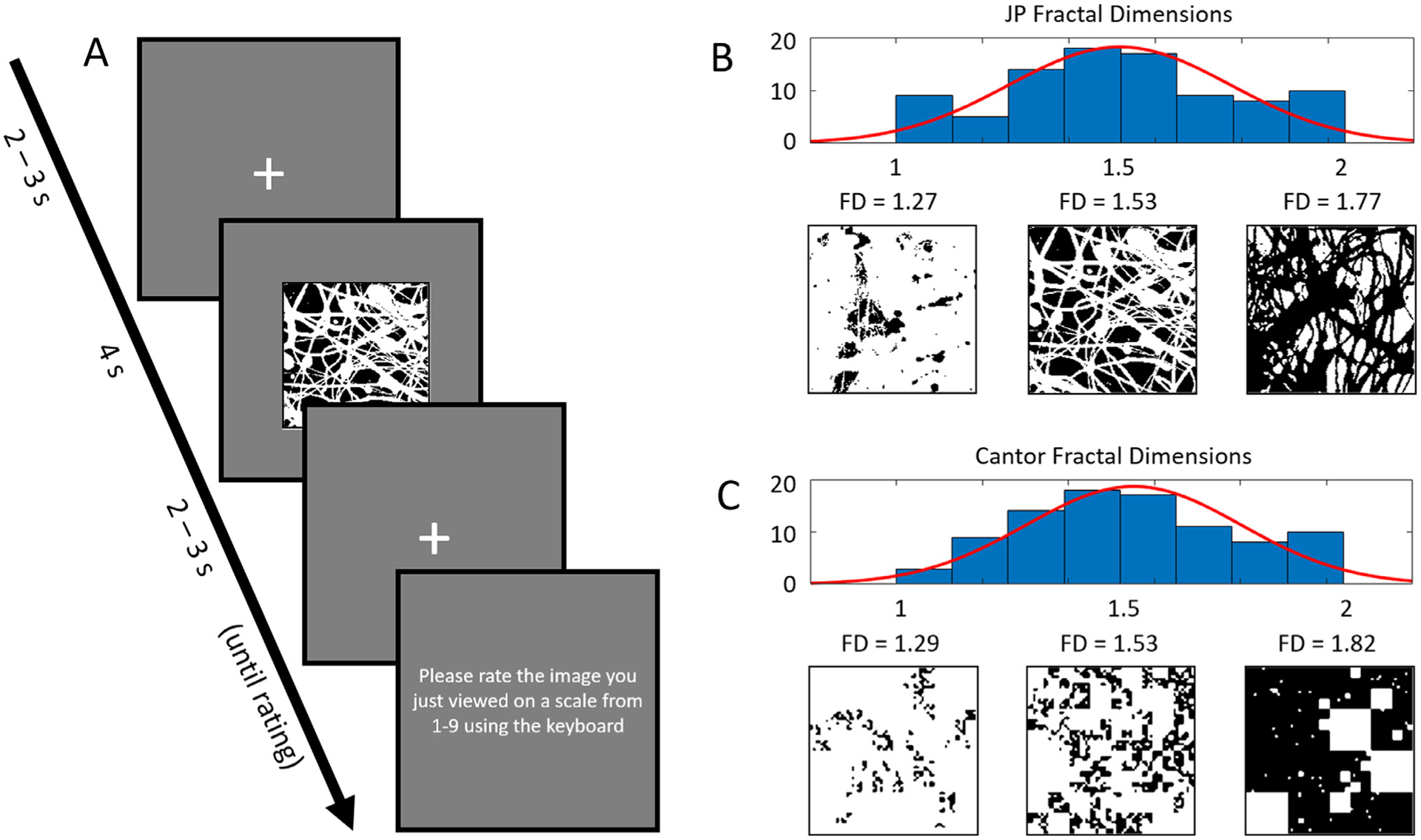
A: Timing and design of the fractal pattern viewing and rating task (pattern in diagram is scaled up for visibility). B: Distribution of fractal dimensions for the Jackson Pollock (JP) white layers employed in the current study with three example patterns. C: Distribution of fractal dimensions for the random Cantor sets employed in the current study with three example patterns.

Each pattern was a two-dimensional fractal pattern stimulus that was either derived from a binarized layer of a Jackson Pollock (JP) drip painting or developed algorithmically as a two-dimensional random Cantor set. White layers of drip paintings were provided by Taylor, R.P. as used in (Taylor et al., 2007, 2011). The full process of extracting these fractal layers from scans of the artwork is beyond the scope of the article, as it is not our own original work; we refer the reader to (Taylor et al., 2007, 2011) for an in-depth description of the layer extraction process. Briefly, each art piece consists of multiple different colored layers. Separation of each differently colored layer was performed by identifying the RGB range for that layer and filtering the scanned artwork accordingly, then binarizing the resulting filtered image. This procedure resulted in several layers from each piece of art, each with their own respective fractal dimension. As described in (Taylor et al., 2007), these layers together span a wide range of fractal dimensions between 1 and 2, and as such are well-suited for use as stimuli in a parametric design as employed here. Random Cantor sets were generated as a set of 1s that were divided into subsets, and each subset multiplied by 0 with probability *p*. Thus, this produces a binary “dust” pattern with white features and black “holes” in the dust. The fractal dimension of the resulting pattern can be controlled by changing the probability value *p*. Fractal dimensions of the images were calculated using the drip dimension (DD) statistic as described in Taylor and colleagues (2011), therefore accurately characterizing low-dimensional fractality resulting from drip layers in Jackson Pollock paintings. This method computes the common box-counting dimension across a range of box sizes for each image, and determines a local minimum of the log-log slope of the resulting box size × dimension function, with calculated DD ranging from 1.10 – 1.89. See Fig. 1 for a diagram of the pattern viewing task, fractal image statistics, and examples of the stimuli used for the current study.

### 2.3. EEG recording and processing

Continuous EEG data were recorded using a Biosemi ActiveTwo system (Biosemi B.V., Amsterdam, The Netherlands). Sixty-four sensors were mounted in an elastic cap, positioned according to the International 10/20 system. Vertical eye movements were recorded from two channels placed immediately beneath each eye (VEOG), and horizontal eye movements were recorded from two channels placed at the outer canthi of the eyes (HEOG). Unlike most other EEG systems, the Biosemi system measures the reference-free voltage between each sensor and a common sense mode (CMS) sensor, and all referencing is accomplished offline.

Continuous data were imported into Matlab and processed using EEGLAB 14 (Delorme & Makeig, 2004). Data were rereferenced to linked mastoids, low-pass filtered at 35 Hz (transition band: 30.625 – 39.375 Hz; chosen to avoid filtering out any activity below 30 Hz) using a zero-phase FIR filter, downsampled to 125 Hz with anti-aliasing, and high-pass filtered at .6 Hz (transition band: .3 − .9 Hz; chosen to avoid filtering out any activity above 1 Hz) using a zero-phase FIR filter. Bad channels were detected and removed using EEGLAB functions; channels were removed if the joint probability of the channel given the observed data was more than 3 standard deviations from the expected probability. Bad channels were not interpolated before running Independent Component Analysis (ICA). Data were epoched into 5 second trials surrounding pattern presentation (−1 second before to 4 seconds after). Infomax ICA (Makeig et al., 1996) was computed on the epoched data.

As our study included a long stimulus viewing time, and participants were encouraged to thoroughly inspect each stimulus, we used strict criteria to ensure cleaning of ocular artifacts from our data. This was accomplished using several procedures, including the use of ADJUST to detect ocular artifacts with four different measures: 1) spatial average distances, 2) spatial variance differences, 3) maximum epoch variances, and 4) spatial eye differences, measures which were found in Mognon et al. (2011) to have high levels of effectiveness for removal of ocular artifacts from the EEG. We applied additional criteria in SASICA to optimize detection of ocular artifact, via correlation of component timecourse with H/VEOG channel timecourses. As our recording setup used two vertical and two horizontal EOG sensors, SASICA automatically used the differences of H/VEOG channels to increase the signal-to-noise ratio for detection of saccade components in the EEG. The application of SASICA and ADJUST together was shown in (Chaumon et al., 2015) to detect nearly 100% of saccades in each training dataset, and as such is currently considered state-of-the-art in detection and rejection of eye movement components in the EEG. Additionally, since muscular artifact can appear as higher-frequency EEG activity (Muthukumaraswamy, 2013), we detected ICA components with low temporal autocorrelation and removed them, as this has been shown to be particularly sensitive for detection of muscular artifact in the EEG. Detected artifactual components were removed. Data were then epoched into 4-second non-overlapping windows surrounding presentation of each pattern (0 seconds before to 4 seconds after). The epoch mean was removed from individual epochs, and remaining artifacts were detected as epochs containing voltage values +− 125 microvolts. Finally, channels that were previously removed were spherically interpolated.

### 2.4. EEG power spectral analysis

EEG were analyzed using power spectral analysis. One-sided single-trial power spectra were calculated using Welch’s method (MATLAB *pwelch()* function). We used default window and overlap parameters (8 segments with 50% overlap), and a 250-point discrete Fourier transform (providing frequency resolution of 0.5 Hz). Data were converted to power spectral density (PSD) using a decibel transform (10log10(*data*)), and frequencies from 1 to 30 Hz (in 0.5 Hz steps) were returned for further analysis. All PSD analyses were conducted on the resulting within-subject channel × frequency × trial matrix of PSD values.

### 2.5. Single-trial analysis of behavior and brain responses to fractal patterns

As EEG data frequently exhibit departures from normality, in particular at the single-trial level (Cohen, 2014), and since our rating data were rank-ordered, we used non-parametric Spearman correlations for our single-trial analyses of ratings and EEG-PSD. While previous single-trial analyses have generally used Spearman correlations, it is possible that the relationship between fractal dimension and preference might be nonmonotonic, and thus not adequately captured by Spearman correlations. However, in all cases we found that Spearman correlations provided a better description of the data than a quadratic regression (S1). Group-level significance of within-subject single-trial Spearman correlations was analyzed by applying *t*-tests (with multiple comparison correction, in the case of PSD data).

Behavioral analysis was conducted within single subjects to assess whether individual preferences were related to pattern fractal dimension. Within each subject, we calculated Spearman correlations between the fractal dimension of the image and the rating of preference the subject assigned to the image. This was done separately for the JP and Cantor image stimuli. We then tested the between-subject significance of these correlations (after normalization via Fisher’s z-transform) using two one-sample *t*-tests. We also compared within-subject correlations between fractal dimension and preference using a paired-sample *t*-test. Finally, given previous reports that there might exist subgroups of individuals with different fractal dimension-aesthetic preference relationships (Bies, Blanc-Goldhammer, et al., 2016; Spehar et al., 2016; Street et al., 2016), we tested for this possibility by clustering individuals on the basis of within-subject correlations between fractal dimension and rating (two observations per subject). Note that we clustered on within-subject correlations because our sample size could not support clustering directly on ratings for stimuli of different fractal dimensions (180 total observations) (Dolnicar et al., 2014). For this analysis we fit Gaussian mixture models (GMMs; latent profile analysis) for one and two components to the dimension-preference correlation data. The best-fitting model was selected using the Bayesian Information Criteria (BIC) (Schwarz, 1978).

Single-trial analyses of power spectral density (PSD) EEG were also conducted within individual subjects. For each point (sensor × frequency), Spearman correlations were computed across trials between single-trial EEG power spectra and fractal dimension. To facilitate condition comparisons, correlations were run separately for JP and Cantor images. This resulted in four sets of Spearman correlations for each participant (JP dimension, JP rating, Cantor dimension, Cantor rating), which were normalized via Fisher’s z-transform prior to mass univariate significance testing. Significance of single-trial correlations was assessed by using permutation-based cluster corrected (Maris & Oostenveld, 2007) one-sample t-tests against a null hypothesis of zero correlation, in order to obtain sets of contiguous data points that responded to parametric variations in complexity and rating of fractal images. As an inherent subjective choice in the use of cluster correction is the alpha level at which neighboring points are set to join as a cluster (Maris & Oostenveld, 2007), we used the implementation of threshold-free cluster enhancement (TFCE) described in (Mensen & Khatami, 2013) with 5000 permutations to obtain clusters of significance without any subjective selection of cluster joining threshold. We used this same method to compare single-trial fractal dimension and rating correlation maps obtained for JP and Cantor images using paired-samples *t*-tests. Additionally, since TFCE provides a test value for each point (unlike more traditional testing), we calculated effect sizes for between-subject EEG-PSD significance tests (Cohen’s d).

## 3. Results

### 3.1. Image statistics

A Wilcoxon rank-sum test indicated that fractal dimensions did not significantly differ between JP and Cantor images, *z* = −0.95, *p* = .34, confirming that our comparison images were properly matched in their fractal dimensions. To rule out other potential low-level confounds in our results, for each image we also calculated a summary statistic describing the distribution of energy across spatial frequencies according to (Eskicioglu & Fisher, 1995). We confirmed that within each type of image, distribution of spatial frequencies was uncorrelated with fractal dimension (JP images: *p* = .22, Cantor images: *p* = .12). As all of our primary analyses used the fractal dimension of each image as the primary variable of interest, this confirms that our results cannot be explained by a systematic relationship of spatial frequency with fractal dimension. We note that all images were also implicitly controlled for contrast, Michelson contrast (Michelson, 1927) = 1 for all images, since all images were black-and-white.

### 3.2. Single-trial behavioral results

**Fig. 2.**
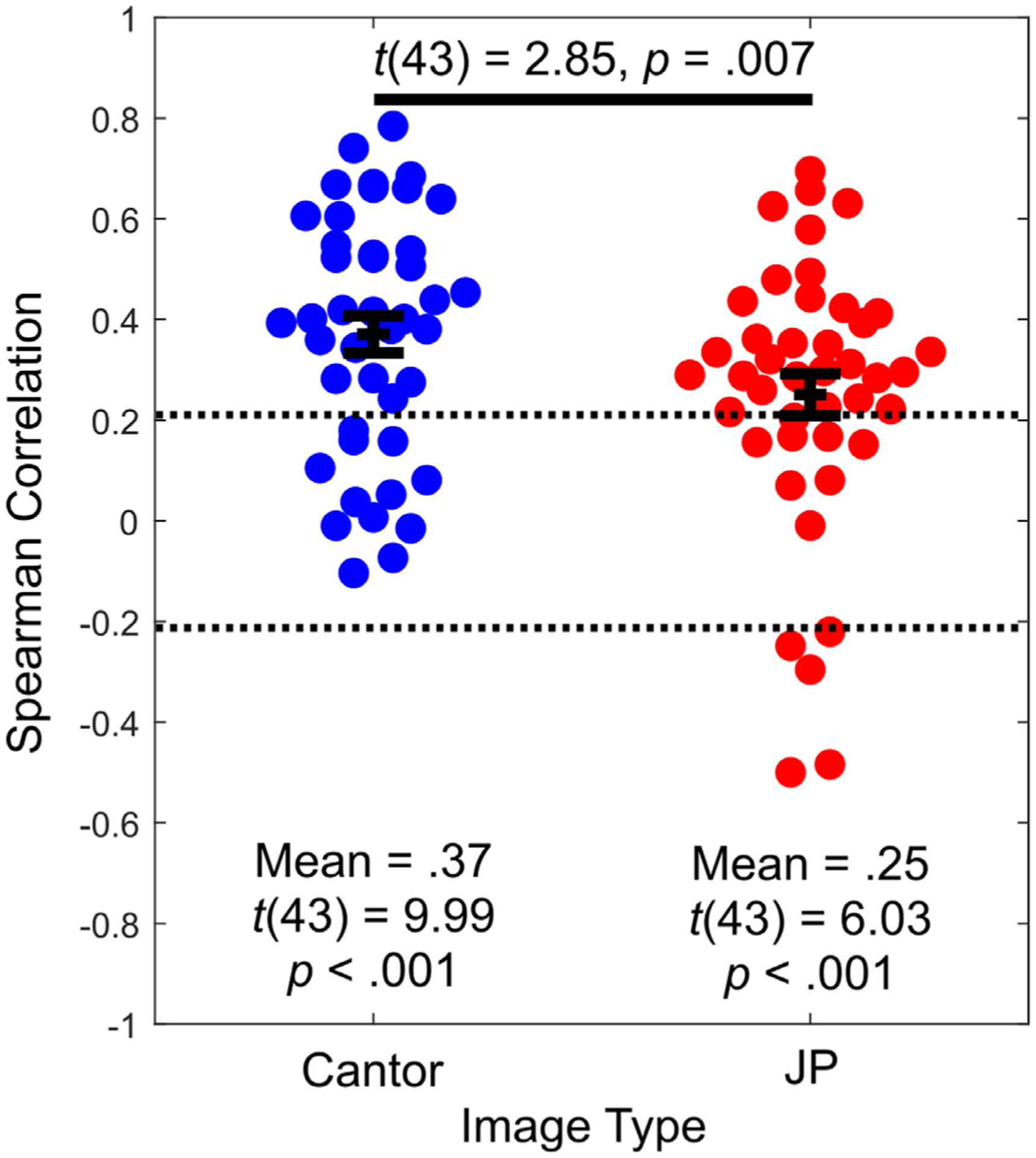
Swarm plots of within-participant Spearman correlations between the fractal dimension and subjective preference (plotSpread.m function, https://www.mathworks.com/matlabcentral/fileexchange/37105-plot-spread-points-beeswarm-plot). Points represent single-subject correlations of fractal dimension and rating, error bar represents ±1 SEM. Horizontal spread of points indicates the density of observations at that level of y (akin to a vertical histogram, or a violin plot). Dotted lines represent within-subject significance level (Spearman rho = ± .207).

Results of single-trial Spearman correlations indicated that for both JP and Cantor images, most subjects rated higher fractal dimension images more favorably than lower fractal dimension images (Fig. 2), and correlations were significantly non-zero and positive for both JP and Random Cantor images (both *p* < .001). See Fig. 2 for a summary of the single-trial relationships between fractal dimension and preference ratings, including within-subject correlation values and group-level *t*-tests for significance. Across subjects, the fractal dimension-rating correlation was stronger for Cantor images, compared to JP images (*p* = .007). We also calculated a Spearman correlation between the (within-subject) correlation coefficients for each type of image. This result indicated that across subjects, within-subject correlations between fractal dimension and rating were positively correlated (Spearman’s rho = .47, *p* = .001); as such, subjects that preferred high-D JP images also tended to prefer high-D Cantor images. Latent profile analysis indicated that a one-component model provided the best fit to our data (S1), and as such we proceed in the remainder of our analysis without dividing participants into subgroups.

### 3.3. Single-trial EEG correlates of fractal dimension perception

Single-trial power spectral analysis of EEG was used to examine frequency-specific representations of fractal dimension for both JP and random Cantor images. For JP images, increasing fractal dimension correlated with decreased alpha power predominately over left occipital and parietal sensors (maximal at sensor O1, 14 Hz, *t*(43) = −5.41, TFCE-corrected *p* = .001), an effect that was associated with a large effect size (Cohen’s d = 0.82). Increasing fractal dimension of JP images also correlated with decreased beta power, predominately over occipital and parietal sensors (maximal at sensor P8, 21 Hz, *t*(43) = −5.15, TFCE-corrected *p* = .004), an effect that was associated with a medium-large effect size (Cohen’s d = 0.77).

**Fig. 3.**
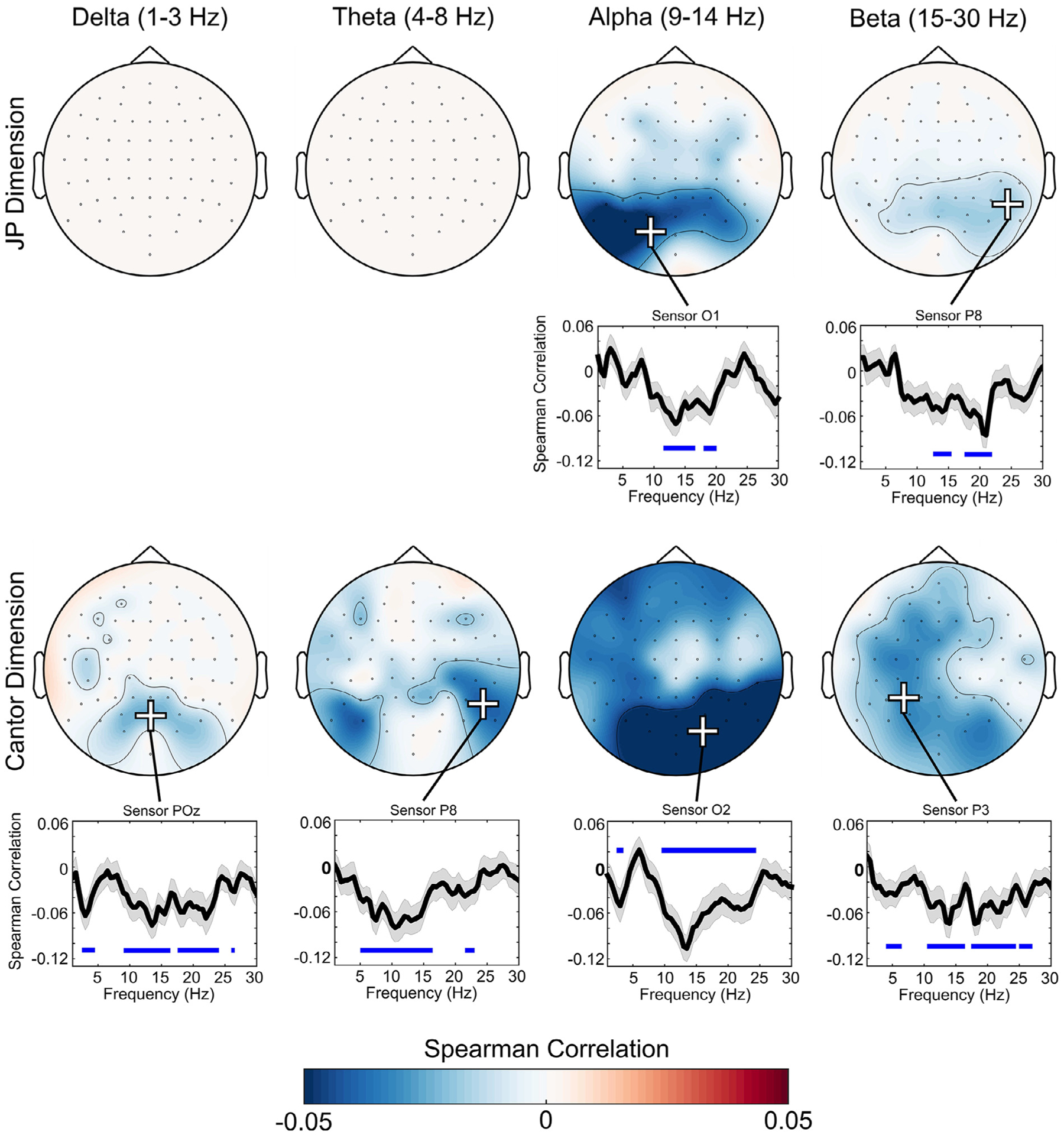
Plots of significant PSD-fractal dimension correlations. Topographic plots are shown threshholded at TFCE-p < .05. Line plots show correlation coefficients at sensors with maximal effects (marked by + signs on topographic plots), as determined quantitatively using TFCE. Red shading on line plots indicates ± 1 SEM, and blue horizontal lines indicate regions with TFCE p < .05 (i.e. regions of significance).

For random Cantor images, we found a broadband decrease in spectral power with increasing fractal dimension. This effect was present in delta, theta, alpha, and beta frequency ranges, and was most prominent over parietal-occipital sensors for all frequencies. Delta effects were maximal at sensor POz (3 Hz, *t*(43) = −3.43, TFCE-corrected *p* = .017), and were associated with a medium effect size (Cohen’s d = 0.52). Theta effects were most prominent at sensor P8 (7 Hz, *t*(43) = −3.91, TFCE-corrected *p* = .003), and were associated with a medium effect size (Cohen’s d = 0.59). Alpha effects were maximal over sensor O2 (13.5 Hz, *t*(43) = −6.18, TFCE-corrected *p* < .001), and were associated with a large effect size (Cohen’s d = 0.94). Beta effects were most prominent over sensor P3 (18 Hz, *t*(43) = −4.81, TFCE-corrected *p* = .003), and were associated with a medium-large effect size (Cohen’s d = 0.72). Single-trial PSD-fractal dimension correlations did not significantly differ for JP and Cantor images, all *p* > .1. See Fig. 3 for a summary of PSD-fractal dimension correlations, and see Fig. 5 for a summary of effect sizes.

### 3.4. Single-trial power spectral correlates of fractal image preference

**Fig. 4.**
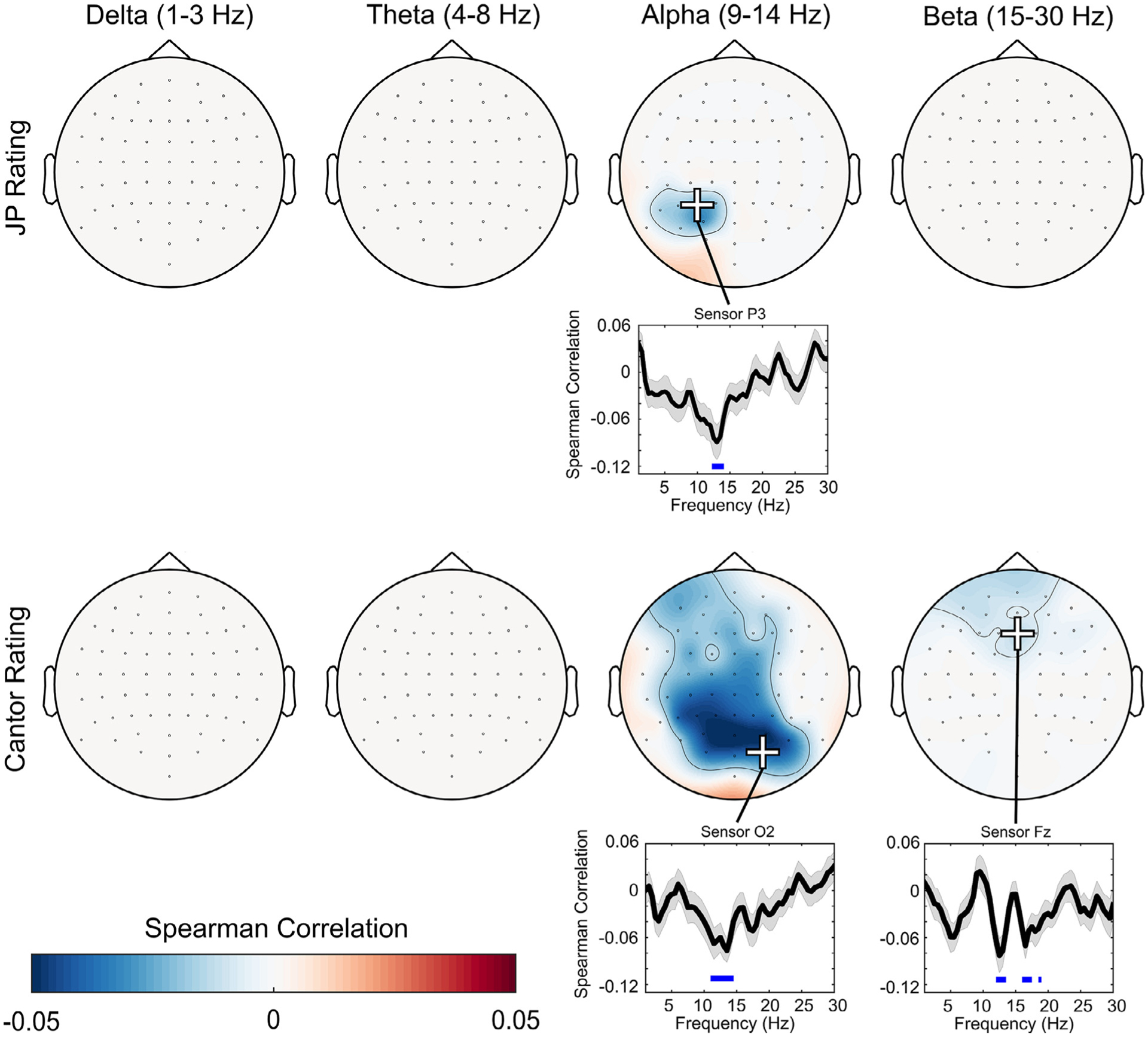
Plots of significant PSD-preference correlations. Topographic plots are shown threshholded at TFCE-p < .05. Line plots show correlation coefficients at selected sensors with maximal effects (marked by + signs on topographic plots), as determined quantitatively using TFCE. Red shading on line plots indicates ± 1 SEM, and blue horizontal lines indicate regions with TFCE p < .05 (i.e. regions of significance).

**Fig. 5.**
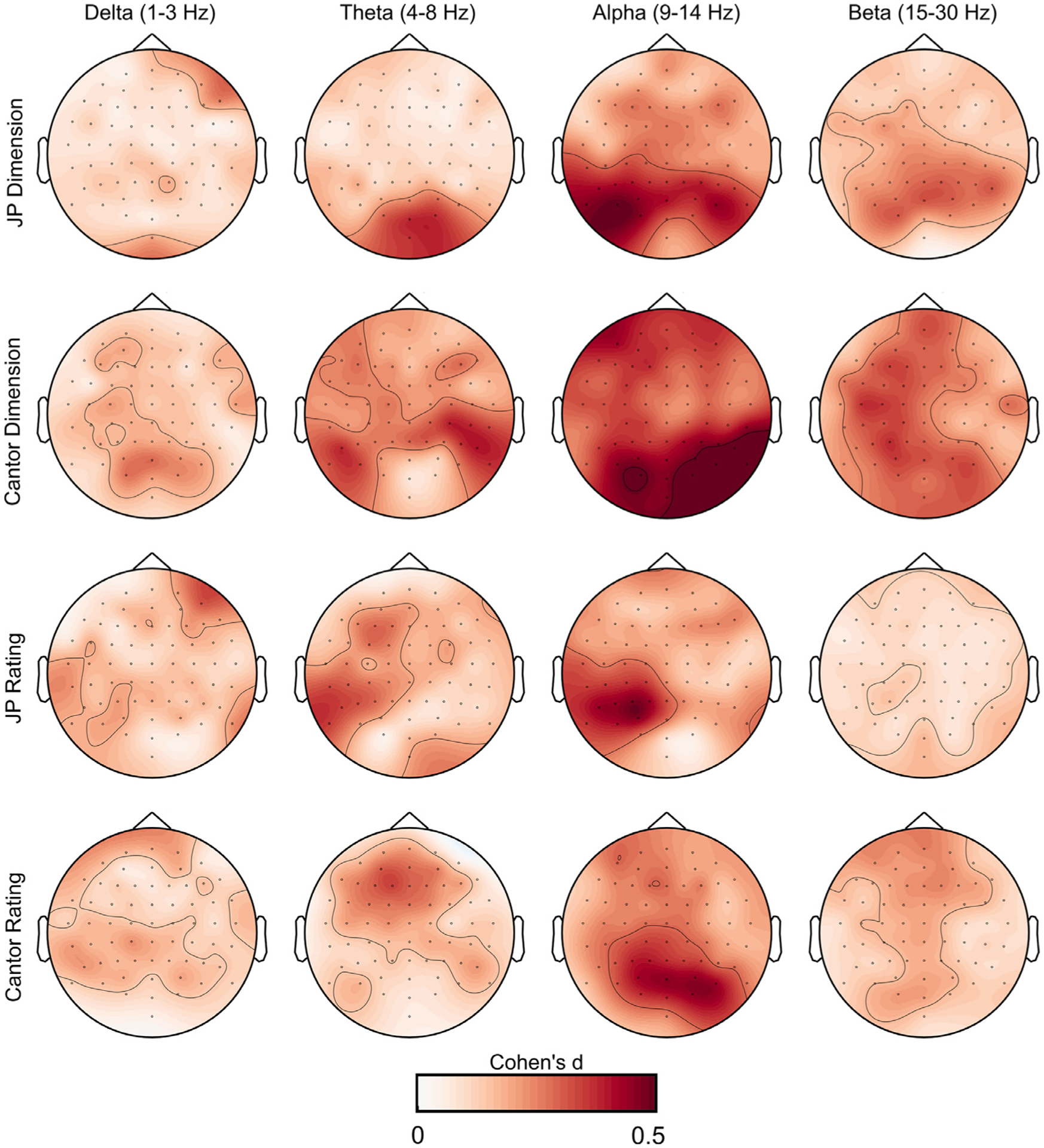
Topographic plots of effect size (Cohen’s d) for each EEG-PSD analysis reported.

Single-trial EEG power spectral analysis was used to probe frequency-specific representations of aesthetic preference for both JP and random Cantor images. For JP images, only power at alpha frequencies correlated significantly with preference for fractal patterns. This effect was maximal over sensor P3 at 13 Hz (*t*(43) = −4.13, TFCE-corrected *p* = .036), and was associated with a medium effect size (Cohen’s d = 0.62). For random Cantor images, alpha frequencies correlated with preference ratings over a cluster of parietal channels (maximal at sensor O2, 13.5 Hz, *t*(43) = −5.15, TFCE-corrected *p* = .006), an effect that was associated with a medium-large effect size (Cohen’s d = 0.78). Beta frequencies correlated with preference ratings over a cluster of frontal channels (maximal at sensor Fz, 16.5 Hz, *t*(43) = −3.59, TFCE-corrected *p* = .023), an effect that was associated with a medium effect size (Cohen’s d = 0.55). Single-trial PSD-preference correlations did not significantly differ for JP and Cantor images, all *p* > .1. See Fig. 4 for a summary of PSD-preference correlations, and see Fig. 5 for a summary of effect sizes.

## 4. Discussion

In the present study, we sought to understand electrophysiological brain activity in response to artistic and mathematical fractal patterns. Participants were presented with either artistic fractals (Jackson Pollock [JP] white layers) or statistically matched random Cantor fractals, while recording electrical activity with EEG. After presentation of each image, participants were asked to rate each image in terms of personal preference. Behavioral results indicated that subjects preferred higher fractal dimension (D) stimuli to lower D stimuli, for both JP fractals and matched random Cantor sets, and whole-scalp EEG analysis revealed that occipital-parietal alpha and beta activity was modulated by fractal complexity of the patterns, with power decreasing as complexity increased. Furthermore, we found that parietal alpha power parametrically tracked personal preference for JP images and for random Cantor sets, with decreased parietal alpha power predicting increased subjective preference. The recovered average Spearman correlations using EEG-PSD have maximal magnitudes of ~0.08–0.10, which are generally in line with those reported in similar single-trial correlation analyses (Cavanagh, 2015; Rawls & Lamm, 2021), and the effects were found to be highly reliable and associated with medium-to-high effect sizes (Cohen’s d for one-sample t-tests). As such, we present evidence that fractal complexity and preference are coded parametrically by EEG power spectra during pattern viewing.

### 4.1. Subjective preference of fractals

As D value increased, preference ratings increased. This correlation was greater for the random Cantor sets, but was significant and positive for the JP images as well. Patterns of within-subject correlations between fractal dimension and preference were themselves correlated, indicating that subjects who preferred high-D JP stimuli also preferred high-D Cantor stimuli. This result is interesting, as there is disagreement in the literature as to whether participants prefer high-dimension fractals or mid-dimension fractals. For example, one study found no relationship between fractal dimension and subjective preference when participants viewed fractal landscapes, but once images of water or hills were removed from the set there was a striking positive correlation between complexity and preference (Hagerhall et al., 2004) (although note that these images only covered the range from D = 1.1–1.5, and it is thus unclear whether this result can be extrapolated to higher frequency ranges). As none of our image sets included water or hills, it is possible that this debate hinges largely on the content of the images being viewed rather than a strict preference for certain fractal dimensions. Other studies have shown that ratings of exact fractals increased monotonically with higher D (Bies, Blanc-Goldhammer, et al., 2016). This study also found that a small number of participants preferred low-D fractal patterns. We also found that overall, participants preferred higher-D fractal patterns, while a small portion of participants instead preferred lower-D patterns. While latent profile analysis did not support the existence of subgroups (S1), it is possible that a larger sample would allow for the separation of a low-D preferring subgroup.

A limitation of our analysis of preference for fractal patterns is that the single-trial framework only allowed us to make conclusions about the presence or absence of monotonic relationships in the data. Future analyses might extend the single trial framework to include analysis of nonlinear within-subject relationships, which would allow for detection of nonlinear relationships such as preference for mid-range fractals compared to low- or high-D fractals. This analysis could presumably be done using nonlinear within-subject regression, although to our knowledge this procedure has never been applied to EEG data. We do, however, note that we compared the fit of a quadratic regression to the fit of Spearman correlations for the preference data, and we found that Spearman correlations describe the fractal dimension – preference relationship more accurately. Future methodological improvements might fit more detailed within-subject models to assess preference for fractal patterns. There is also an open question regarding the best method for assessing subjective preference of viewed images. Our rating scheme used a Likert-type scale, which is one of the most preferred methods for assessing preference, while some studies instead used two-alternative forced-choice ranking. This difference might explain some of the discrepancies between our results and prior analyses. However, for the wide variety of fractal dimensions we assessed, a two-alternative forced-choice task would have required an impossible number of trials, so studies using two-alternative forced-choice procedures would have to either be inordinately long or use a much smaller variety of stimuli than our study used.

### 4.2. Single-trial eeg results

We found that alpha and beta power were negatively modulated in parietal regions with respect to increasing fractal complexity for both image types (artistic or mathematical), suggesting a shared mechanism for visually processing details of an image. Intriguingly, this activity occurred over similar frequencies for both Cantor and JP stimuli; that is, for both stimulus types, alpha and beta PSD correlated negatively with fractal dimension. This suggests that overall, higher-complexity imagery is associated with decreased parietal alpha and beta power, in line with evidence that alpha activity might be suppressed during periods of higher attention. Simulations suggest that gamma activity emerges in the context of local excitatory/inhibitory interactions, while beta activity subserves longer-range neuronal communication (Kopell et al., 2000). Aissani and colleagues (2014) proposed that the beta activity could code for mid-level processes in the visual hierarchy (e.g. depth ordering, computation of border ownership, contour completion, and filling in surfaces), and deep cortical layers that receive feedback from distant regions frequently produce more beta activity (Donner & Siegel, 2011).

Our finding that higher D values resulted in less beta power is in line with the evidence showing that beta power is indicative of long range communication between populations of neurons, because the intricate detail of these images likely relies on more local, short range neural communication (e.g. line/edge detectors), while the less complex images allow for higher-level interpretations of aspects like depth ordering and contour completion. These results are also broadly in line with those presented in (Hagerhall et al., 2008), whose authors indicated that low-D fractal patterns produced lower alpha and beta power in compared to high-D fractal patterns. However, our results are not strictly comparable to those in (Hagerhall et al., 2008), due to differences in the manner of EEG frequency analysis and the parametric design and analysis employed in the current manuscript.

Beta synchronization also modulates other perceptual processes, such as perceptual switches in binocular rivalry, with low beta synchronization correlating with holding a consistent perceptual state, while a significant increase in beta power occurs during perceptual switches (i.e. while integrating a new percept) (Piantoni et al., 2010). The higher beta power at lower D values could be attributable to the ambiguity of the image, as the observer actively explores possible interpretations of bistable contours in the object recognition process, given that low to mid D values induce object pareidolias at a higher rate than more complex D values (Bies et al., 2016). Conversely, low alpha and beta at high D values may reflect active visual processing, as well as the inherent perceptual stability of an image that contains intricate details too complex to give rise to multiple interpretations. Future research will examine our interpretation of the role of cortical alpha and beta modulations in reports of object pareidolia; if this interpretation is correct, it is expected that fractals generating lower parietal alpha and beta power will also result in fewer reported pareidolias.

Though the discovered parametric relationship between alpha/beta power and fractal complexity is intriguing, an important limitation should be noted. The complexity level of the stimuli used in the present study is correlated with the number of black pixels in the image. Given that brightness may affect parietal and occipital activity (Eroğlu et al., 2020), further neuroscience research with stimuli that can be modulated to control for overall luminance is needed.

### 4.3. Brain-behavior relationships: Neural correlates of subjective preference

Using our single-trial parametric analysis of EEG power spectra, we were able to identify consistent neural correlates of preference for both JP and random Cantor images. For both image types, increased preference correlated with decreased alpha power in occipital and parietal regions. As such, we suggest that alpha power may indicate the degree to which a subject finds an image aesthetically pleasing. Previous research suggests that alpha power is related to both creative processes and turning attention inwards (Benedek et al., 2014). Additionally, alpha power is reliably expressed by the default mode network (DMN) (Knyazev et al., 2011), which is associated with mind wandering and self-referential thought (Buckner et al., 2008). Furthermore, the DMN is consistently activated by paintings rated most “aesthetically moving” with respect to each individual (Vessel et al., 2012, 2013). Thus, alpha power found in relation to fractal pattern preference (JP and Cantor) could reflect participants actively turning attention inwards to relate the image to themselves. Furthermore, increasing complexity of fractals (JP and Cantor), and increasing ratings of these fractals, both correlated with decreased alpha power. This suggests that similar cortical regions engaged by pattern complexity are related to aesthetic preference, providing intriguing initial evidence of a brain mechanism that might instantiate the theorized direct relationship between complexity and aesthetic preference (Birkhoff, 1933).

A limitation of these results putatively describing a neural correlate of preference for complex fractal patterns must be noted. There is a strong relationship between dimension and aesthetic preference for images. Our analyses also indicate substantial overlap between neural power spectra coding for preference and for complexity of fractal patterns. As such, there is limited evidence for neural power spectral correlates specific to personal preference, and there remains the possibility that parietal alpha power tracks primarily fractal complexity. This might be the case, as previous work has indicated that choice preference for patterns might instead be reflected in frontal alpha power (Chew et al., 2016). Thus, while we provide some evidence that fractal dimension and preference might be coded by parietal alpha power, future studies should aim to orthogonalize measures of preference and dimension so that neural correlates of dimension perception and preference can be independently analyzed. Additionally, we note that there are many different types of fractals. As such, these are only a few possible explanations of these results and future work should clarify neural activity underlying aesthetic response to other classes of fractals, such as strange attractors (Aks & Sprott, 1996) and Brownian motion images (Bies, Boydston, et al., 2016).

### 4.4. Conclusion

We present a detailed analysis of whole-scalp EEG PSD patterns influenced by the complexity of fractal images. We used a set of fractal art patterns, derived from Jackson Pollock paintings (Taylor et al., 2007, 2011), and a well-matched set of random Cantor sets with the same fractal dimension distribution as the artistic fractals. For both artistic and mathematical random fractal patterns, subjective preference increased for higher fractal dimensions (D), representing more complex patterns. Increasing D also correlated with lower alpha and beta power over parietal sensors. Furthermore, preference for both artistic fractals and for random Cantor fractals correlated with parietal alpha. This is the first parametric (rather than categorical) analysis of EEG frequencies during fractal pattern viewing, as previous analyses have grouped fractals into discrete categories, while fractal dimension is in reality a continuous variable. Future work should build on these results by examining nonlinear relationships between brain activity, fractal dimension, and aesthetic preference, as well as the relationship of visual pareidolias to fractal dimension, preference, and EEG activity.

## Supplementary Material

1

## Figures and Tables

**Table 1:** Self-reported gender, age, and race for included and excluded participants.

Demographics	Options	Total N	Included	Excluded	Included-Excluded Difference
**Gender**	M	29	26	3	*χ*^2^(1) = 1.06, *p* = .41
	F	19	15	4	
	NR	3	3	0	
**Race**	White	37	33	4	*χ*^2^(4) = 4.80, *p* = .27
	Black/African-American	3	2	1	
	Asian/Asian-American	5	3	2	
	Multiracial/Other	3	3	0	
	NR	3	3	0	
**Age**	Mean	20.04	20.02	20.14	*t* (46) = 0.13, *p* = .90
	Standard Deviation	2.20	2.32	1.46	

NR = not reported.
