# Supplementary material for: Parametric Cortical Representations of Complexity and Preference for Artistic and Computer-Generated Fractal Patterns Revealed by Single-Trial EEG Power Spectral Analysis
Source: Neuroimage. Author manuscript; Available in PMC 2021 Aug 1. (PMC8287964; doi:10.1016/j.neuroimage.2021.118092)
Supplement: 1 [file NIHMS1722738-supplement-1.docx]

**SUPPLEMENTAL MATERIAL for:**

**Parametric Cortical Representations of Complexity and Preference for Artistic and Computer-Generated Fractal Patterns Revealed by Single-Trial EEG Power Spectral Analysis**

Eric Rawls^1^*, Rebecca White^2^*, Stephanie Kane^3^, Carl E. Stevens, Jr.^3^, Darya L. Zabelina^3^

^1^Department of Psychiatry and Behavioral Sciences, University of Minnesota Health

^2^Department of Psychology, University of New Hampshire

^3^Department of Psychological Sciences, University of Arkansas

In this supplement to the main manuscript, we present the results of an exploratory Gaussian Mixture Model that we fit to explore the possibility of subgroups with different fractal pattern preference in our full sample. Additionally, we display the results of an analysis indicating that Spearman’s rank-order correlation better describes the fractal dimension-rating relationship than a quadratic regression.


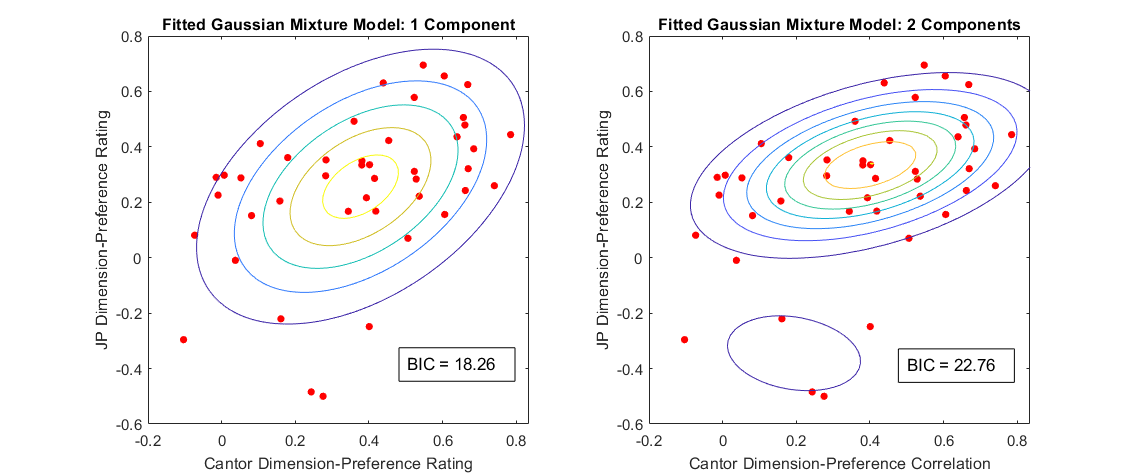


*Supplemental Figure 1. Given previous reports that there might exist subgroups of individuals with different fractal dimension-aesthetic preference relationships (Bies et al., 2016; Spehar et al., 2016; Street et al., 2016), we tested for this possibility by clustering individuals on the basis of within-subject correlations between fractal dimension and rating (two observations per subject). Note that we clustered on within-subject correlations because our sample size could not support clustering directly on ratings for stimuli of different fractal dimensions (180 total observations) (Dolnicar et al., 2014). For this analysis we fit Gaussian mixture models (GMMs; latent profile analysis) for one and two components to the dimension-preference correlation data. The best-fitting model was selected using Bayesian Information Criteria (BIC) (Schwarz, 1978). Latent profile analysis indicated that a one-component model provided the best fit to our data, and as such the remainder of our analysis did not divide participants into subgroups.*


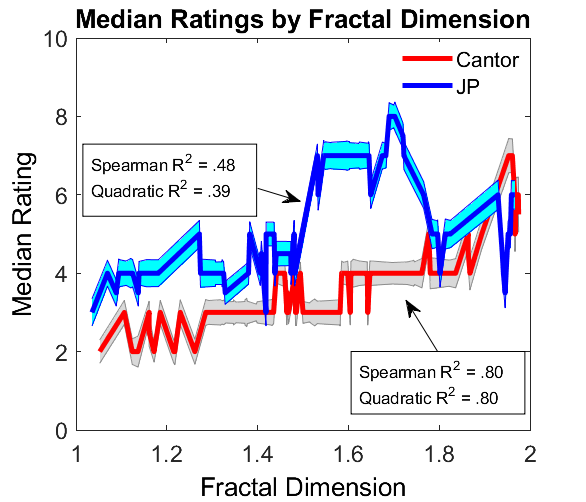


*Supplemental Figure 2. Given the possibility of nonmonotonic relationships between fractal dimension and rating data, we calculated the median rating across subjects for each image and compared the fit of a Spearman correlation to a quadratic regression relating fractal dimension to rating. We found that rank-order correlations explained a greater amount of variance in ratings than quadratic regression for JP images, and fit equally well for Cantor images. This supports our primary use of Spearman correlations in our within-subject analyses, as previously used in single-trial analysis.*

**Supplement References**

Bies, A. J., Blanc-Goldhammer, D. R., Boydston, C. R., Taylor, R. P., & Sereno, M. E. (2016). Aesthetic Responses to Exact Fractals Driven by Physical Complexity. *Frontiers in Human Neuroscience*, *10*. https://doi.org/10.3389/fnhum.2016.00210

Dolnicar, S., Grün, B., Leisch, F., & Schmidt, K. (2014). Required Sample Sizes for Data-Driven Market Segmentation Analyses in Tourism. *Journal of Travel Research*, *53*(3), 296–306. https://doi.org/10.1177/0047287513496475

Schwarz, G. (1978). *Estimating the Dimension of a Model*.

Spehar, B., Walker, N., & Taylor, R. P. (2016). Taxonomy of Individual Variations in Aesthetic Responses to Fractal Patterns. *Frontiers in Human Neuroscience*, *10*. https://doi.org/10.3389/fnhum.2016.00350

Street, N., Forsythe, A. M., Reilly, R., Taylor, R., & Helmy, M. S. (2016). A Complex Story: Universal Preference vs. Individual Differences Shaping Aesthetic Response to Fractals Patterns. *Frontiers in Human Neuroscience*, *10*. https://doi.org/10.3389/fnhum.2016.00213
